# X-ray inactivation of RNA viruses without loss of biological characteristics

**DOI:** 10.1038/s41598-020-77972-5

**Published:** 2020-12-08

**Authors:** Babak Afrough, Jonathan Eakins, Sarah Durley-White, Stuart Dowall, Stephen Findlay-Wilson, Victoria Graham, Kuiama Lewandowski, Daniel P. Carter, Roger Hewson

**Affiliations:** 1 National Infection Service, Public Health England, Porton Down, SP4 0JG UK; 2 Centre for Chemical, Radiation and Environmental Hazards, Public Health England, Chilton, OX11 0RQ UK; 3 CBR Division, Defense and Science Technology Laboratories, Porton Down, SP4 0JG UK; 4 Faculty of Infectious and Tropical Diseases, London School of Hygiene and Tropical Medicine, Keppel Street, London, WC1E 7HT UK

**Keywords:** Biological techniques, Microbiology

## Abstract

In the event of an unpredictable viral outbreak requiring high/maximum biosafety containment facilities (i.e. BSL3 and BSL4), X-ray irradiation has the potential to relieve pressures on conventional diagnostic bottlenecks and expediate work at lower containment. Guided by Monte Carlo modelling and in vitro 1-log_10_ decimal-reduction value (D-value) predictions, the X-ray photon energies required for the effective inactivation of zoonotic viruses belonging to the medically important families of *Flaviviridae*, *Nairoviridae*, *Phenuiviridae* and *Togaviridae* are demonstrated. Specifically, it is shown that an optimized irradiation approach is attractive for use in a multitude of downstream detection and functional assays, as it preserves key biochemical and immunological properties. This study provides evidence that X-ray irradiation can support emergency preparedness, outbreak response and front-line diagnostics in a safe, reproducible and scalable manner pertinent to operations that are otherwise restricted to higher containment BSL3 or BSL4 laboratories.

## Introduction

Climate change, increases in population density, deforestation and urbanization are altering the geographical range of vectors that transmit dangerous viral pathogens, so promoting the spillover and emergence of zoonotic diseases into new regions and placing immunologically naïve populations at risk^1,2^. A significant proportion of these emerging zoonosis are RNA viruses which present characteristically high transmission rates, are subject to unpredictable evolutionary change and cause high-consequence infectious diseases (HCIDs) in humans. Some notable examples include avian influenza (AI), Ebola virus (EBOV), severe acute respiratory syndrome coronavirus (SARS-CoV), West Nile virus and Zika virus (ZIKV) which have all caused unprecedented epidemics across several countries, and in many instances death^3^ or congenital disease^4^, while exerting acute pressures on public health infrastructures and local economies^5^. Incidences for such HCIDs are predicted to increase as population density, urbanization and climate equilibria continue to fluctuate^6–10^. These relatively recent events, including the current emergence of COVID-19^11,12^ highlight the need for effective and reproducible workflows that permit safe, scalable and rapid pathogen inactivation, suitable for a range of downstream pathogen detection and intervention strategies.

Established methodologies for virus inactivation include chemical^13–15^, thermal^16^, ultraviolet^17^, plasma^18^, electron bombardment^19^ and radiological sources^20,21^ which can all have either selective or limited downstream applications. X-ray irradiation is an alternative to chemical and thermal approaches which both have known detrimental effects on the biological integrity of samples^22–24^. It does not require the addition of exogenous toxic or volatile chemicals and does not induce notable thermal expansion within the sample. In contrast to UV, plasma and electron bombardment, high energy X-rays (≥ 1 keV) are composed of highly penetrative photons that traverse multiple layers of packaging in a similar manner to gamma radiation. Compared to gamma radiation, however, X-ray sources do not suffer from radio-isotopic half-life decay or the same security implications that are associated with the use of ‘live’ radiological sources. Energy-spectra and dose rates are also more tunable with X-rays than with gamma sources.

The damage caused by photons is a consequence of target matter ionization and the disruption that this entails for the biological system, both directly via molecular break-up and indirectly via subsequent ionization and endogenous free-radical chemical reactions. At X-ray energies from ~ 1 keV up to a ~ few 10 s keV, photoelectric absorption is the primary interaction, in which the photon is absorbed by the atom and an electron is ejected. From a ~ few 10 s keV to a ~ few 10 s MeV, Compton scatter dominates, in which an electron is ejected but the photon is not absorbed and can hence cause additional ionization elsewhere in the sample. Above ~ 1.25 meV, electron–positron pair production processes can occur, and at higher energies still photonuclear, photo-disintegration and other ‘exotic’ processes can begin to arise^25^. In all cases, relaxation processes within the ionized atom (e.g. Auger and Coster-Kronig electron emission, fluorescence photons etc.) further contribute to the transmission of energy within the sample. The precise energy ranges above which one process dominates over another depend on the effective atomic number (*Z*) of the material^26^. The amount of energy transferred from the field to the material is quantified by the absorbed dose, defined as the energy deposited in a given volume divided by its mass^27^.

The probability that a photon interacts with a given atom generally decreases with increasing energy^28^, suggesting that lower energy X-rays are likely to be more efficient than more penetrating X-ray or gamma sources in the context of biological inactivation. However, and as an inevitable consequence, photon penetration decreases with decreasing energy, leading to potential dose gradients within samples. This implies that determination of the optimum X-ray energy for biological inactivation of macroscopic samples is non-trivial. Soft X-rays (1 to 10 keV) have been shown to have some value in the inactivation of both enveloped^29^ and non-enveloped viruses^30^ without causing excessive damage to antigenic properties as previously demonstrated using pathogenic bacterial models^31,32^.

This study defines the amount and type of X-ray radiation required to produce replication deficient ZIKV (genus *Flavivirus*), Hazara virus (HAZV, genus *Orthonairovirus*), Bebaru virus (BEBV, genus *Alphavirus*) and Rift Valley Fever virus (RVFV, genus *Phlebovirus*), and provides D-values to achieve their respective controlled inactivation. The viruses selected here span a range of genomic arrangements and physical structural variants which are surrogates for higher risk group viruses that cause HCIDs e.g. Omsk hemorrhagic fever, Crimean-Congo Hemorrhagic Fever and Chikungunya fever diseases. Using D-values defined here, evidence of comparable inactivation efficacy of X-rays to gamma radiation with retention of key biochemical and immunological characteristics is presented, for use in downstream applications requiring high-fidelity nucleic acid or protein substrates.

## Results

### Low-energy X-rays produced by bremsstrahlung are responsible for virus inactivation efficacy

To characterize the energy distributions of the photon fields for virus irradiation, Monte Carlo (MC) models of the X-ray source and irradiation chamber environment fitted to a MultiRad 225 Irradiator were developed using the general-purpose radiation transport code MC N-Particle (MCNP) version 6.1^33^, designed to track many particle types over broad ranges of energies. Setting the tube potential to 220 keV and current to 18.2 mA within the model, X-ray photon energies and dose rates for the irradiation of liquid virus samples through multi-layered packaging were modelled using an unfiltered beam, and 0.2, 0.5 or 2.0 mm aluminum (Al) or 0.3 mm copper (Cu) filters. The dose rates were calculated by the use of ‘kerma tallies’ (average kinetic energy released per unit mass), with equivalence between absorbed dose and kerma assumed; this assumption was justified from a consideration of the physical geometry of the irradiation chamber and sample set-up, and the maximum expected ranges^34^ of any secondary electrons that might be produced. Conversion to absolute dose rates from the relative ‘per-source-electron’ normalization applied by default by MCNP6.1 was achieved by multiplying the Monte Carlo results by the quotient of the beam current of the X-ray tube (typically 18.2 mA, or equivalently 18.2 mC s^-1^) divided by the elementary charge constant (~ 1.602 × 10^–19^ C).

**Figure 1:**
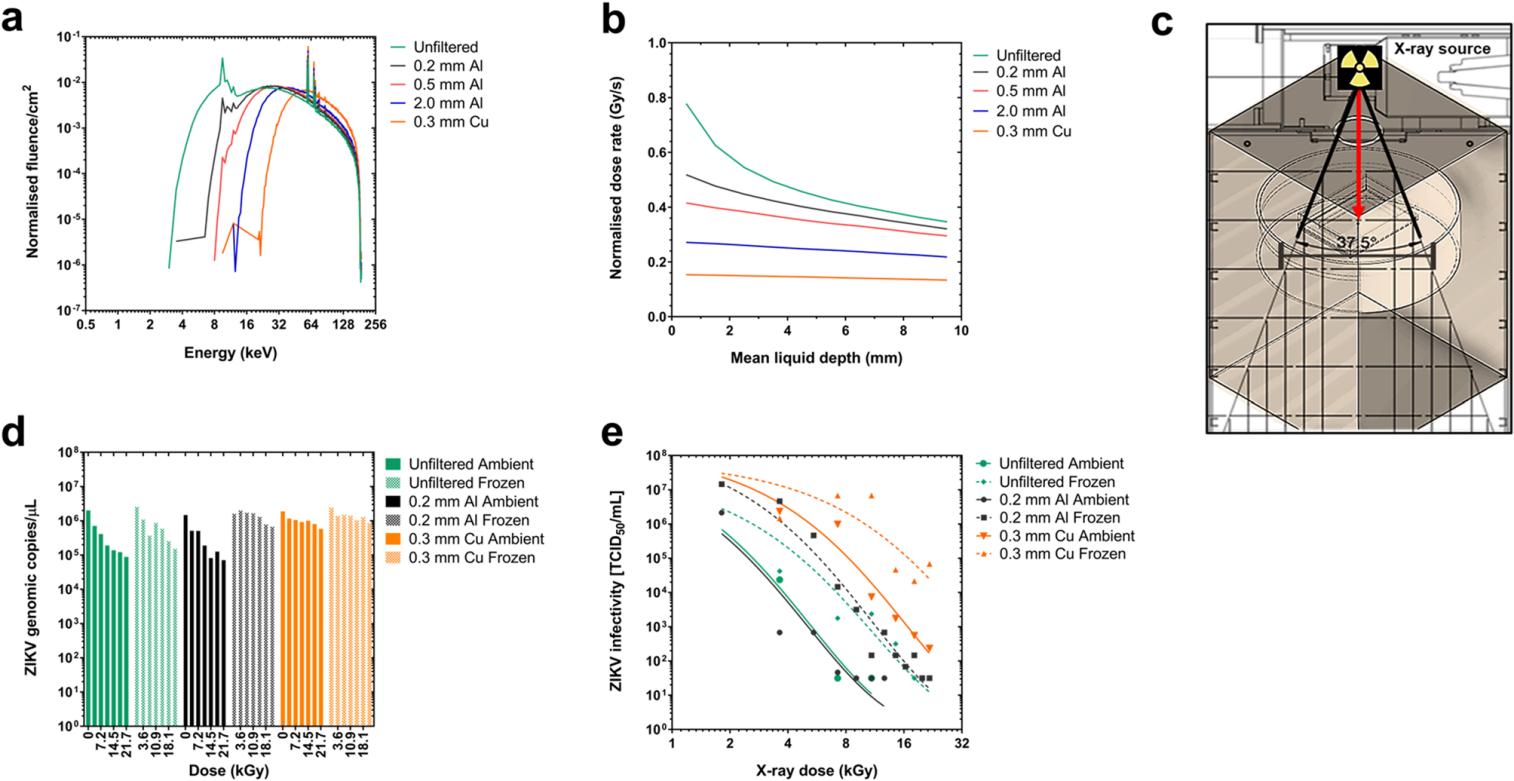
Effect of X-ray beam filtration on ZIKV inactivation. Monte Carlo simulation of beam filtration effect on (a) X-ray photon energy spectra showing beam hardening^58,59^ and (b) dose-to-depth penetration of photons in water at 220 keV, 18.2 mA using MCNP6.1 model in (c) the irradiation chamber showing the sealed virus specimens with packaging dimensions of 10 cm × 8 cm × 1 cm (L x W x D) at a distance of 22.6 cm within a 37.5° irradiation cone produced by the MXR-225/26 tube. Incremental X-ray irradiation of ZIKV at the above conditions showing (d) the effect of beam filtration on ZIKV RNA genomic-equivalent copies using RT-qPCR and (e) Infectivity titer of ZIKV by TCID_50_ assay. All data was acquired by accounting for a sample-to-source distance of 22.6 cm and each data point represents the mean of triplicate independent runs.

Average air kerma values, deduced through MC modelling of predicted free-in-air doses to sealed biological pathogens, were benchmarked against experimental data measured using a TN31010 Semiflex ionization chamber, and they showed a good correlation^35^. MC modelling data at 22.6 cm from the target sample illustrated the presence of a significant bremsstrahlung continuum beginning at ~ 10 keV with the expected characteristic peaks at ~ 60 keV and ~ 70 keV from *L*, *K*_*α*_ and *K*_*β*_ shell transitions in the X-ray source tungsten target. The systematic filtration of the radiation beam using the above metals with increasing mass-thicknesses resulted in the removal of low-energy bremsstrahlung radiation but maintained the high energy characteristic radiation (Fig. 1a). The data in Fig. 1a were obtained via the use of MCNP6.1 ‘*f4:p*’ photon fluence tallies, binned into 0.5 keV increments, that were defined over small volumes of air located just below the position of the filter and were self-normalized to the total integrated fluence recorded by that tally over all energy bins.

To account for beam attenuation and loss of dose rate during photon penetration at an increasing liquid depth of sample, absolute dose rates as a function of depth were calculated using the same modelling parameters. For these calculations, the representative 1 × 1 × 1 cm^3^ volumes were subdivided into a set of 10 horizontal slices of thickness 1 mm, and a MCNP6.1 ‘*f6:p*’ photon kerma tally was defined over each layer; renormalization to the 18.2 mA applied beam current of the X-ray tube was then applied to obtain absolute dose rates from the relative Monte Carlo data. Doses in the first few milliliters of water were found to be significantly larger than those at greater depth. The data in Fig. 1b demonstrate that dose gradients are greater for an unfiltered field, due to higher intensities of poorly penetrating low-energy photons, but conversely less for more filtered fields, which are proportionally harder.

Our MC data of photon energies and beam penetration through the sample packaging predicted an average dose rate of 0.496 Gy/s (std. dev. ± 0.075) in the geometric field of the X-ray beam (SI Appendix Fig. S1a, b and c) and supported confidence in dose uniformity and virus sample exposure levels during radiation cycles. Figure 1c illustrates a diagram of the experimental setup including the distance from the modelled X-ray source to the virus samples. These sets of data taken together provide key parameters for reproducible inactivation of infectious material and can be used to scale inactivation conditions in a reliable manner.

Using ZIKV as an example, the effect of beam hardening by filtration on virus inactivation were examined using two endpoint readouts of absolute genomic quantification by reverse transcriptase real-time PCR (RT-qPCR) and viral infectivity using the 50% tissue culture infectious dose (TCID_50_) assay. Beam hardening experiments were carried out with frozen and liquid/physiological (ambient) sample conditions. D-values, defined as the radiation-dose required to reduce the viral infectivity titer by 90% (or 1-log_10_) were determined by the method of Schmidt and Nank^36^ using TCID_50_ data.

Filtration with 0.3 mm Cu led to the greatest reduction in low-energy bremsstrahlung photons (or soft X-rays) and exposed the samples to fields more dominated by the characteristic X-rays. This led to a reduced efficacy in ZIKV inactivation, as measured by RT-qPCR (Fig. 1d) and TCID_50_ data (Fig. 1e). Unfiltered beam exposure produced a similar inactivation curve to that from a beam filtered with 0.2 mm Al but showed higher variability, causing inconsistencies across ZIKV inactivation. This data set in combination with predictions obtained from the MC modelling data rationalized the use of 0.2 mm Al for beam filtration to ensure a better balance between dose uniformity and dose rate attenuation in all subsequent inactivation experiments.

The highest efficacy of viral inactivation was achieved using 0.2 mm Al filtration under ambient conditions for ZIKV (D-value 1.83 kGy); beam filtration with 0.3 mm Cu showed a significantly higher D-value (2.94 kGy), suggesting that it is the low-energy component of the photon field (below ~ 20 keV) that is the most effective for viral inactivation, in turn suggesting a photoelectric mechanism of inactivation. In the context of sample conditions (i.e. frozen or ambient), a similar trend to ZIKV was observed when comparing frozen RVFV (3.84 kGy) and ambient (2.63 kGy) conditions using 0.2 mm Al beam filtration. To this end, 0.2 mm Al filtration under ambient conditions at 220 keV, 17.5 mA were subsequently used to produce inactivation curves and determine D-values for the selected viruses in this study. All doses were identified through pre-set time-dependent radiation exposure experiments while absorbed dose was monitored in real-time. From here on, viruses treated with X-rays are denoted with the suffix “-X” (*e*.*g*. ZIKV-X) with the related exposure dose in kGy indicated in superscript.

### D-values predict non-infectious ZIKV and RVFV in vitro and in vivo

Following the determination of D-values, ZIKV and RVFV sterility were assessed and confirmed via in vitro viability experiments. All inactivation doses were examined for cytopathic effects (CPE) by three consecutive rounds of tissue culture passages in Vero cells. Total virus input for all irradiated samples were controlled using RT-qPCR readouts to ensure that a minimum of 10^8^ genomic-equivalent copies/mL of X-ray treated virus (pre-irradiation infectious titer of 10^6^ TCID_50_/mL) were added to each cell culture monolayer. This provided assurance that the development of any CPE in vitro during the assay was normalized across the X-ray treated samples throughout the course of the experiment. As expected, non-irradiated ZIKV and RVFV controls developed CPE in Vero cells at 4 and 3 days post-infection respectively. A correlation between the time of delay before CPE and the X-ray dose was observed throughout the three-week assay.

**Figure 2:**
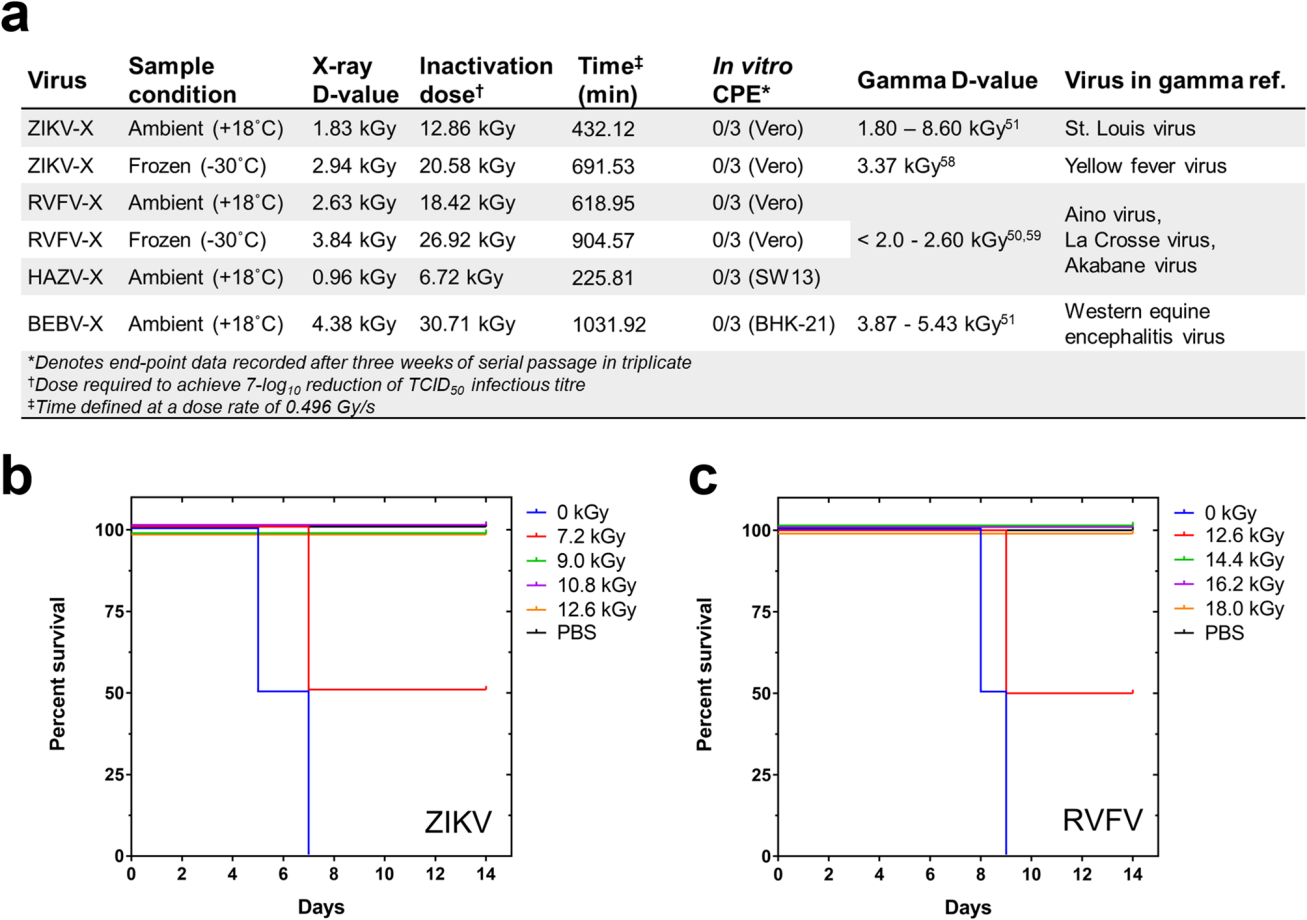
Inactivation of viral pathogens using X-rays. (a) X-ray D-values showing the dose required to inactivate 1-log_10_ of virus at 220 keV, 17.5 mA with 0.2 mm Al filtration. In vitro CPE was not observed in an infection-sensitive cell substrate after treatment with 1 mL of virus and following 3 passages at predicted inactivation doses. X-ray D-values were then compared to gamma inactivation data produced under similar sample conditions, for members of the same genus. (b) ZIKV infectivity in A129 mice after intravenous administration of irradiated ZIKV and (c) RVFV infectivity in BALB/c mice after intramuscular administration of irradiated RVFV; data shows 100% survival of mice for receiving viruses that had been irradiated with inactivating doses of X-rays at predicted D-values, in agreement with in vitro passage data in the appropriate cell line model.

Consistent with the D-values predicted from the inactivation curves, a final dose of 12.8 kGy for ZIKV-X and 15.79 kGy for RVFV-X as judged by CPE assays produced inactivated virus (Fig. 2a). To investigate sterility further, in vivo animal disease models for ZIKV and RVFV were employed to provide additional evidence of non-infectious virus using the predicted X-ray inactivation doses. For ZIKV, interferon type-II deficient A129 mice were intravenously administered with an equivalence of 1 × 10^6^ pfu of irradiated virus (at serial X-ray dose increments) and doses ≥ 9 kGy showed a 100% survival rate at 14 weeks post-infection (Fig. 2b). No significant clinical symptoms or shifts in animal weights were observed during this period of experimentation, whereas mice treated with non-irradiated ZIKV (1 × 10^5^ pfu) and doses ≤ 7.2 kGy consistently presented with clinical symptoms and were euthanized (according to pre-set disease end-points and ethical standards) within six days post-infection. Similar experiments were performed for RVFV using BALB/c mice with similar results: all mice survived after 2 × intramuscular administration of 5 × 10^5^ pfu of inactivated virus (i.e. virus pre-irradiated with doses ≥ 14.4 kGy; Fig. 2c). The organs of surviving mice, including blood, spleen, brain and liver were checked for the presence of viral genomic material using RT-qPCR and were found to be negative, indicating that the RVFV-X treated with doses ≥ 14.4 kGy were no longer replicating and were not infectious in BALB/c mice. D-values for HAZV and BEBV were found to be 0.96 and 4.83 kGy respectively.

It was observed that in vitro viability systems are a more sensitive method for determining loss of viral replication as it permits the sampling of a larger amount of virus for CPE. However, in vitro and in vivo results display a good correlation and re-affirm the predictive model of D-values for inferring virus sterility after X-ray irradiation.

### Biochemical and immunological characterization of X-ray inactivated virus

**Figure 3:**
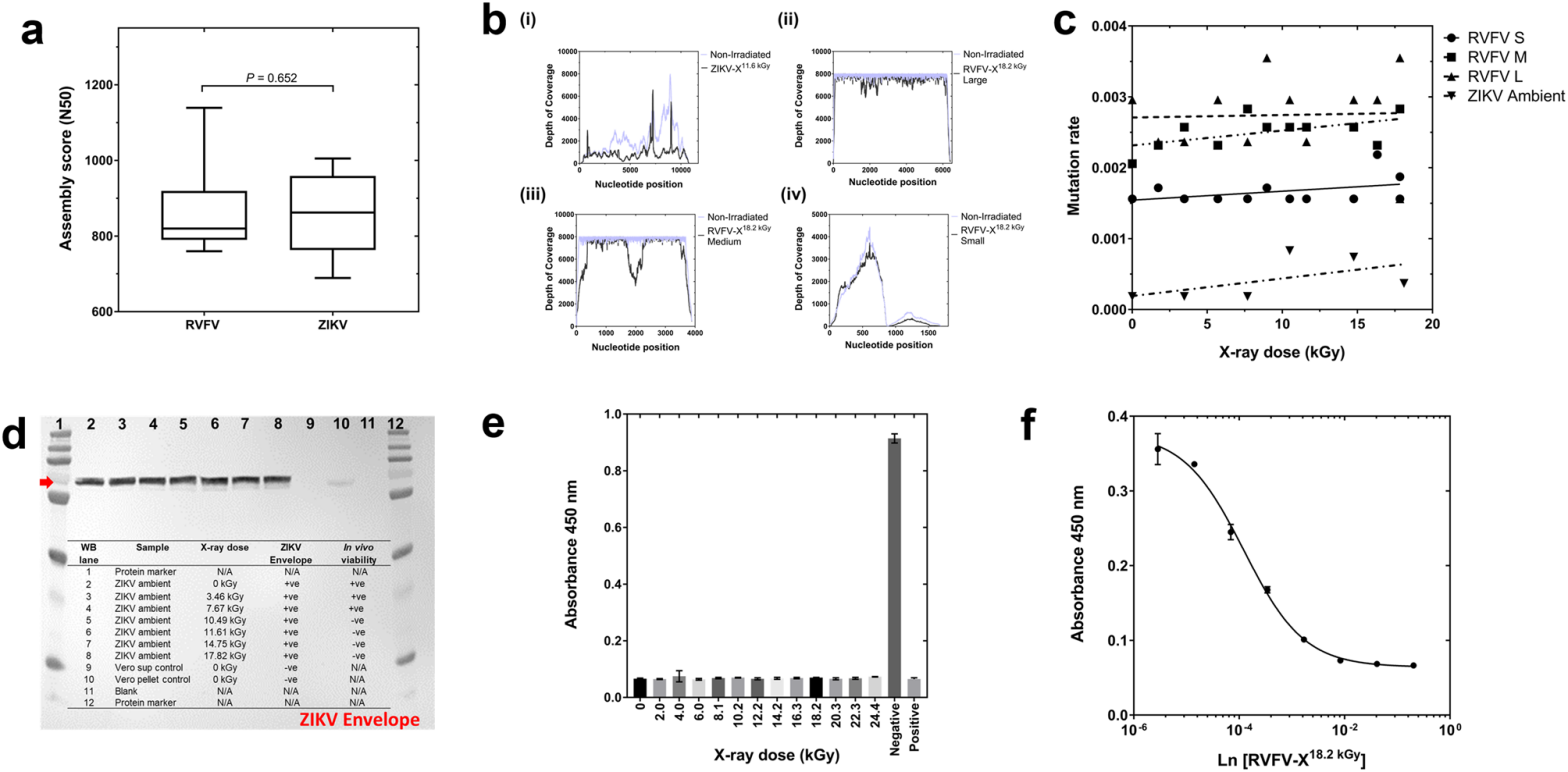
Nucleic acid and protein analysis of X-ray inactivated virus. (a) Box plot of N50 contig assembly statistics for RVFV and ZIKV, showing no statistical differences (Mann–Whitney U, two-sided) between viral contig re-assembly across radiation doses (0 to 24 kGy), (b) NGS mapping data demonstrating similar depth of coverage when comparing sequencing of non-irradiated and irradiated ZIKV-X and RVFV-X and (c) regression analysis of variants induced through X-ray irradiation across dose increments, showing non-significant correlations (*r*^2^ ≤ 0.32 ). The effect of irradiation on viral proteins by (d) Western blot detection of ZIKV envelope illustrating uniform sensitivity of detection, (e) abrogation of competition ELISA signal of RVFV anti-NP using X-ray irradiated antigen (n = 3 per dose). (f) two-fold serial dilutions of whole-virus RVFV irradiated with 18.2 kGy of X-rays inhibits the binding of anti-NP in commercially sourced competition ELISA.

An assessment of whether any of the X-ray inactivated viruses could be used as a source of viral RNA/cDNA or protein reagents in downstream biochemical and/or immunological assays was next investigated. To this end, ZIKV-X^11.6kGy^ and RVFV-X^18.4kGy^ were utilized in a series of experiments involving Next Generation Sequencing, ELISA and Western blot analysis. Using virus stocks cultivated at ≥ 6 log_10_ infectious units, the utility of X-ray irradiation under ambient conditions for sequencing and full-length assembly of non-replicating ZIKV-X^11.6kGy^ and RVFV-X^18.4kGy^ genomes using an Illumina paired-end sequencing library was performed. ZIKV has a positive-sense single-stranded RNA genome whereas RVFV consists of a tripartite negative-stranded RNA genome consisting of Small (S), Medium (M) and Large (L) segments. Using SPAdes^37^, it was possible to re-assemble the complete genomes of both ZIKV-X and RVFV-X. Genome assembly showed no significant statistical association between the N50 variance and increasing X-ray irradiation dose, for either virus (Fig. 3a).

Complete ZIKV-X and RVFV-X S, M and L genomes could be successfully reconstructed from two independent non-replicating, X-ray inactivated virus stocks without the need for genome scaffolding. To assess for potential mutations incurred from X-rays during inactivation, complete genome assemblies from the non-irradiated stocks of both viruses were used in the subsequent mapping of irradiated sequencing reads. Mapping experiments to the respective viral genomes showed a minimum of ×500 depth of coverage (Fig. 3bi–iv) for both viral species across all X-ray irradiation doses. Mutations were assessed by calling variants at a coverage depth of ≥ ×100 and Phred quality score 30, against the final assembly of non-irradiated ZIKV and RVFV genomes. Figure 3C shows genomic segments of both inactivated ZIKV-X and RVFV-X containing random counts of substitution mutations, interestingly however no correlations were observed between dose exposure and the number of variants per genome segment(s) of either virus. As all viral stocks used in these sets of experiments originated from a single batch, it is unlikely that the observed variants are from variations in viral propagation or cultivation conditions. It is however probable that these variants are a result of either the mutation rate associated with MLV reverse-transcriptase used during cDNA synthesis^38^ or the proof-reading actions associated with Taq polymerase enzyme during NGS library amplification steps rather than X-ray irradiation.

With respect to protein analysis of X-ray inactivated material, ZIKV-X envelope glycoprotein (Fig. 3d) and RVFV-X nucleoprotein (data not shown) were detected by Western blot using commercially sourced polyclonal antibodies at a similar sensitivity to that found for non-irradiated viral extracts. Matched tissue culture supernatants without propagated virus served as negative controls for the antibodies and did not show cross-reactivity with non-viral antigens. Employing an independent approach, a commercially sourced multi-species competition ELISA kit was adapted to assess the protein integrity of X-ray irradiated RVFV. Serum samples were replaced with irradiated virus preparations and crude non-irradiated C6/36 lysate served as negative control. Figure 3E shows that a 1:10 dilution of all irradiated virus preparations effectively inhibited the interaction of anti-NP antibody whereas C6/36 lysate did not (Fig. 3e). To affirm specificity of interaction, serial dilutions of irradiated RVFV treated with an 18.2 kGy dose was used as competition substrate for anti-NP (Fig. 3f).

## Discussion

Zoonotic infections have been the predominant source of dangerous emerging disease over the last century^9,39^ and include EVD, CCHF, Lassa fever, RVF, MERS and SARS. Work with such zoonotic pathogens requires the use of specialized high-containment facilities necessary for the safe handling of infectious materials. A consequence of the stringent bio-safety features of these facilities, especially BSL4, is a constraint to high-throughput processing which in turn slows the progress of diagnostic reference virology, including R&D work. While many microbiological activities can be accommodated within BSL4, there are significant practical and cost benefits of being able to work outside of containment by effectively inactivating pathogens. However, standard and established methods of virus inactivation such as heat treatment or chemical cross-linking with formaldehyde destroy and/or modify complex protein structures and can lead to decreased sensitivity or artefactual results. This is especially problematic if inactivated pathogens are being used for clinical diagnosis and reference virology. A potential solution is to inactivate with X-rays which does not destroy native antigenic structure and provides a useful route to delivering non-infectious reagents for diagnostics and R&D work in lower containment laboratories and provides comparable results to non-inactivated native material. The production of non-infectious material containing whole-virus characteristics is a distinct advantage as it supports more complex experimental strategies than can be achieved with chemically modified or heat-denatured inactivated viral material. A number of studies examining the parameters that influence direct or indirect mechanisms of viral inactivation such as, sample types and suspension matrices^40–44^, physiological temperature states^45^, particle size and infectious titer^46^ have all indicated that X-ray irradiation could rapidly advance and strengthen conventional public health laboratory activities, and should therefore be considered as a serious contender to the current gold standard radiological sources such as Cobalt-60 for agents requiring BSL3/BSL4 facilities. However, to our knowledge, a detailed study with accurate D-values showing evidence of inactivation of viruses that retain biological and immunological characteristics using X-rays has not been thus far demonstrated. Guided by MC modeling, the data reported here shows the amount and type of X-ray radiation required for effective inactivation of viruses belonging families that cause medically-important zoonotic infections in humans. It was possible to recover nucleic acid for sequencing post inactivation and the protein moieties were detected in routine laboratory analysis.

X-rays offer unique advantages over conventional gamma ray inactivation as they permit higher levels of control over the delivery of beam uniformity to a pathogenic specimen. Although gamma sources such as Cesium-137 or Cobalt-60 can offer very high dose rates compared to the output of typical X-ray tubes, they have significant drawbacks, such as highly divergent fields and inherent difficulties in controlling dose rates and inactivation. Furthermore, gamma source security, shielding, decommissioning and management introduce a range of addition non-scientific problems which are not simple to address. In contrast, X-ray sources are more controllable and directional, permitting improved accuracy in the dose uniformity ratio and in the inactivation process, consequently they reduce the risk of unnecessary damage to the material. This resolution in uniformity can be controlled using choice filtration composites with appropriate attenuation properties for lower energy photons. This is an important factor in the context of developing and scaling X-ray inactivated viruses for rapid diagnostics, high resolution structural biology or potentially for emergency vaccines which can be rapidly inactivated^47–49^ to support front-line healthcare workers in HCID outbreaks.

Initial experiments focused on determining the optimum dose required for a 1-log_10_ reduction in virus titer under frozen and ambient (18˚C) conditions. A comparison of the two conditions showed that protein and nucleic acid characterization could be achieved in both cases. However, physiological conditions permitted quicker inactivation by a few orders of magnitudes as defined by D-values, consistent with previous inactivation reports using gamma and X-ray irradiation of viruses in different matrices including those containing high protein concentrations or in animal tissues such as brain and blood^20,50–53^.

Filtration permits the removal of soft X-rays (≤ ~ 20 keV) by a reduction of bremsstrahlung continuum photons in the X-ray beam. In our experiments looking at the effect of filtration on the irradiation of ZIKV, 0.3 mm Cu filtration produced inefficient inactivation of virus under both ambient (-3.8 log reduction) and frozen (-2.8 log reduction) sample conditions; this compared to -6.6 log reductions for the unfiltered or 0.2 mm Al filtered X-ray doses. These data taken together, suggest that X-ray energies lying on the hard X-ray electromagnetic spectrum are less responsible for inactivation and that soft X-rays are more accountable for the inactivation of viruses through a specific photoelectric ionization mechanism. Filtration of X-rays using 0.3 mm Cu resulted in a significant loss in dose rate which would have to be compensated for by a longer cycle of irradiation to achieve the required dose. On the other hand, unfiltered X-ray beams showed enhanced variability both in the context of depth-to-dose penetration of X-rays within the liquid phase of the samples as well as across the sample plane. Irradiation under these conditions would result in over-exposure of the samples to achieve assurance that the required dose had been used for sample inactivation. Filtration using 0.2 mm Al permitted the penetration of soft X-rays across the packaging system of our samples and induced sample inactivation with decimal reduction values comparable to the literature (Fig. 2a). Furthermore, 0.2 mm Al filtration permitted uniform exposure of the samples to predicted X-ray doses required for inactivation. These parameters showed the successful inactivation of ZIKV, BEBV, HAZV and RVFV under ambient conditions.

An accurate understanding of doses required for sample inactivation using D-values is useful for normalizing exposure times relative to infectious titer and, in turn, will prevent unnecessary damage to precious samples following prolonged irradiation procedures. These D-values can be achieved with current plug and play instrumentation such as the MultiRad 225 keV, which provides highly penetrative irradiation doses permitting viral inactivation through stringent sample packaging systems. Through selection of composite materials with low X-ray attenuation coefficients or high *Z*-values for sample packaging, it may be possible to focus the desired photon energies responsible for inactivation of the pathogen and reduce irradiation times further. Higher dose rates can be achieved by considering dual or triple X-rays sources which could reduce the exposure time significantly and achieve uniform sample inactivation. The sources are relatively straight forward to operate locally and are much safer than radioisotopes because they do not generate nuclear waste or require such stringent security and protective measures.

In principle, it has been demonstrated here that X-ray inactivation can be successfully achieved for a selection of medically-important zoonotic viruses. X-ray irradiation is an effective mode of pathogen inactivation, which permits the production of non-infectious whole virus material that retains desired biochemical and immunological properties under ambient inactivation conditions. Preparedness in conjunction with active bio-surveillance programs are crucial public health endeavors and X-ray irradiation offers a unique tool for rapidly developing reagents for a range of downstream applications in a safe and reproducible manner.

## Materials and methods

### Virus stocks and cell lines

The following virus-cell systems were used for virus cultivation. Zika virus (ZIKV) African strain MP1751 (NCPV accession 1308258v) was cultured at 37 °C, 5% CO_2_ for 3 days in Vero cells (ECACC accession 84113001) using MEMα media supplemented with 10% fetal bovine serum (FBS). Hazara virus (HAZV) strain JC280 was cultured at 37 °C, 5% CO_2_ for 6 days in SW13 cells (ECACC accession 87031801) using MEMα-10% FBS. Bebaru virus (BEBV) was cultivated at 37 °C, 5% CO_2_ for 2 days in BHK21 clone 13 cells (ECACC accession: 85011433) using MEMα-10% FBS. Rift Valley Fever virus (RVFV) strain ZH501 was cultured at 28 °C for 5 days in C6/36 cells (ECACC accession 89051705) in MEMα-10% FBS supplemented with 1.1 g/L of NaHCO_3_ in the absence of CO_2_. Virus stocks were cultivated in single batches of 150 mL each, using the Nunc TripleFlask system (ThermoFisher, UK) prior to clarification by centrifugation at 6,000 × *g*, 4 °C. Each of the virus species were then aliquoted in to 1 mL working stocks and frozen in preparation for pre-irradiation titration and all subsequent irradiation runs. Titration of virus infectivity pre- and post-irradiation was performed by TCID_50_ titration using the Spearman-Kärber calculation.

### Monte Carlo modelling of X-ray photons

MC simulations were performed using the general-purpose radiation transport code MCNP6.1^54^. The model accounts for X-ray source tube potential and current, distance to samples, geometric field of exposure including packaging materials and densities used during irradiation. The source was modelled as a 2.75 mm radius beam of plane-parallel monoenergetic electrons impinging on an angled target of pure tungsten of density 19.6 g/cm^3^. The beam was orientated along the negative x-axis and was hence perpendicular to the vertical (z) axis of the irradiation chamber. In general, the physics parameters and models used in the Monte Carlo simulations were the defaults applied by MCNP6.1, which are the code’s maximally accurate options in almost all cases; the *MCPLIB84* and *e103* cross-section libraries were used for photons and electrons respectively. In order to improve computational efficiency, however, production of bremsstrahlung photons in the tungsten was artificially enhanced by setting the *BNUM* parameter in MCNP to 10,000, with the code then re-weighting the results automatically to maintain fairness; whilst the exact choices of variance reduction applied in the modelling were chosen ‘by hand’, and may not necessarily be optimal, it is important to emphasize that all tally results were successfully tested for statistical robustness using the packages applied by MCNP6.1 by default. Photon intensities and doses were determined using MCNP *f4* fluence- and *f6* kerma-tallies, respectively, with the latter justified under the reasonable assumption of secondary charged particle equilibrium at the locations of interest.

### X-ray irradiation and inactivation

Commercial X-ray generators typically operate by impinging a beam of electrons onto a high-*Z* target (often tungsten) inside an evacuated tube. As the electrons decelerate within the target, bremsstrahlung photons are emitted with a directional distribution that is dependent on the target material and its shape, as well as on the energy of the incident electrons. A thin window in the X-ray tube allows the photons to escape, with collimation typically added around the window to better focus the direction of the beam and prevent the escape of stray radiation. The energy distributions of the X-ray beams are composed of continuous bremsstrahlung radiation that is dependent on the voltage of the electron beam and characteristic peaks that occur at energies dependent on the material. The overall intensity of the X-ray field is governed by the electron beam current. Beam filtration using metals with increasing attenuation coefficients and/or densities can be used to systematically remove the lower energy components of the photon fields, resulting in beam hardening.

X-ray irradiation was performed on a MultiRad 225 keV X-ray Irradiator (Precision X-rays, USA) fitted with an MXR-225/26 X-ray source (225 kV 4 k W). Irradiation dose–response inactivation curves for ZIKV and RVFV were developed for frozen and ambient (18˚C) temperatures. BEBV and HAZV were inactivated under ambient conditions. All virus data were developed by conducting independent irradiation cycles in triplicate. To achieve this, virus supernatant volumes of 1 mL were heat-sealed within 2 layers of heavy-duty polyethylene plastic sheets (500 gauge thick) and treated with 1.8 kGy incremental doses of X-rays to a final dose of 50 kGy. The D-values were calculated as described by Schmidt and Nank^36^ by calculating the gradient of the linear regression-fitted model as a function of log dose vs. log infectious viral titer ($N-N0$), where $N$ is X-ray treated virus and $N0$ is untreated.

### Sterility of irradiated ZIKV and RVFV in vitro and in vivo

All X-ray treated and non-treated ZIKV and RVFV samples were passaged for 3 rounds in vitro in T-12.5 vessels using Vero cells (1 mL of virus/flask, n = 3 per dose) for 7 days per round. Infectious split transfers of 50% cells and culture supernatant were performed at weekly intervals for a period of 3 weeks to define sterility. CPE were monitored daily. IFN-α/β receptor deficient A129 mice (n = 28, 2 mice per radiation dose) and BALB/cOlaHsd 6–8-week-old mice (n = 28, 2 mice per irradiation dose) were used for ZIKV and RVFV sterility studies respectively. The in vivo work was undertaken according to the United Kingdom Animals (Scientific Procedures) Act 1986. These studies were approved by the ethical review process of Public Health England, Porton Down, UK, and by the Home Office, UK. A set of humane end-points based on clinical manifestation of disease was defined in the protocol of the project license. A129 mice were administered intravenously with 400 µL of irradiated ZIKV or non-irradiated control. BALB/c mice were administered intramuscularly with 2 × 50 µL of irradiated RVFV or non-irradiated control.

### RNA isolation and reverse-transcriptase qPCR

Viral RNA was isolated as per the manufacturer’s instructions using the QIAamp viral RNA isolation kit (Qiagen, UK). All RT-qPCR assays were expressed as genomic copy equivalents using a synthetic RNA control transcript (IDT, UK). Quantification of RVFV RNA in mice brain, spleen and liver tissues in the pathogenesis study were normalized to tissue weight. ZIKV assays were performed as per the Lanciotti assay^55^ and RVFV assays were performed as per the Drosten assay^56^ using SuperScript III One-Step RT-PCR kit (Thermo-Fisher, UK).

### Western blot analysis

A 100 µL of irradiated or control ZIKV at 1 × 10^6^ TCID50/mL were concentrated using AmiconR Ultra 0.5 centrifugal filters (Millipore, UK) at 14,000 × *g* for 30 min and resuspended in 30 µL of lithium dodecyl sulfate (LDS) sample buffer containing 50 mM DTT (Invitrogen, UK). Mock infected Vero cell lysate (pellet and clarified supernatant) served as negative control. Protein electrophoresis was performed using NuPAGE Bolt 12% Bis–Tris Plus Gels, 10-well-10 gels (ThermoFisher, UK) at 200 V for 35 min. Electrophoresed proteins were transferred on to ethanol-activated 0.2 µm PVDF membranes using a wet transfer system at 20 V for 60 min and probed with Zika virus Envelope protein antibodies (GeneTex, USA). HRP secondary antibody was detected with 1-Step Ultra TMB-Blotting Solution (ThermoFisher, UK). Mock infected Vero cell lysate (pellet and supernatant) served as negative control. Blot Images were captured on a white light pad fitted to a G:Box F3 using standard settings of their GeneSys analysis software version 1.7.2 (Syngene, UK). Further details can be found in SI Appendix Fig. S4.

### Next Generation Sequencing

Total nucleic acid was isolated using QIAamp viral RNA Mini Kit (Qiagen, UK) from each irradiated sample as per the manufacturer’s instructions. An aliquot of the total nucleic acid (45 µL) was treated with Turbo DNAse I (Invitrogen, UK) following the manufactures instructions and then purified using an RNA Clean and Concentrator—5 kit (Zymo Research, USA). This material was then used in second strand cDNA synthesis using a random nonamer primer A and amplified using primer B using AccuTaq LA DNA Polymerase (Sigma, UK) as previously described^57^. The PCR conditions were set to 98 °C for 30 s denaturation, 30 cycles of 94 °C for 15 s, 50 °C for 20 s, and 68 °C for 10 min. Amplified cDNA was purified using a 1:2 ratio of AMPure XP beads (Beckman Coulter, USA) and quantified using a Qubit and High Sensitivity dsDNA Kit (ThermoScientific, UK). A 2 × 150 bp paired-end Illumina sequencing library was prepared using the Nextera XT V2 Kit with 1.5 ng of cDNA as the input, following the manufacturer’s instructions. Indices were selected using the Illumina experiment manager and sequenced on an Illumina MiSeq by the Central Sequencing Laboratory, Public Health England.

### Genome assembly and variant calling

ZIKV and RVFV genome assembly for each irradiation condition was performed using SPAdes version 3.13.0. Assembly statistics were assessed using Quast version 5.0.2 and are presented as N50 values representing the length of the shortest contig at 50% of the total genome length for ZIKV and RVFV. Depth of coverage for each irradiation time point was performed using BWA version 0.7.12-r1039 and the respective non-irradiated control virus as a reference (ZIKV strain MP1751 and RVFV strain ZH-501). Final consensus determination for each irradiation time point was performed using Pilon version 1.22 using a k-mer of 15. Variants were called using BCFtools MPileup version 0.1.19 at a depth of 100 and Phred 30.

### Competition enzyme-linked immunosorbent assay

Competition ELISA was performed using ID Screen Rift Valley Fever Competition Multi-species ELISA (ID.Vet, France). This assay allows testing of human/animal sera for antibodies to RVFV nucleoprotein (NP) antigen on a solid-phase surface. A 1:10 dilution of X-ray irradiated whole virus preparation (equivalent to 3.0 × 10^5^ pfu/mL) was pre-incubated with the kits competition reagents conjugated to HRP in place of immune sera. The assay was then developed following the manufacturer’s instructions. To confirm specificity irradiated viral antigen abrogating the ELISA signal, twofold serial dilutions of inactivated RVFV prepared using 18.2 kGy of radiation was pre-mixed with the kits competition HRP reagent. Absorbance was measured at 450 nm. Absorbance signal was plotted against a natural log transformation of twofold serial dilutions to express the relationship of irradiated antigen specificity. All figures and graphs were prepared using GraphPad Prism version 7.05.

## Supplementary information

Supplementary Information.
