# Supplementary material for: X-ray inactivation of RNA viruses without loss of biological characteristics
Source: Sci Rep. 2020 Dec 8;10:21431. doi: 10.1038/s41598-020-77972-5 (PMC7722841; doi:10.1038/s41598-020-77972-5)
Supplement: Supplementary file 1 — Supplementary Information. [file 41598_2020_77972_MOESM1_ESM.docx]

**X-ray inactivation of RNA viruses without loss of biological characteristics**

Babak Afrough^1*^, Jonathan Eakins^2^, Sarah Durley-White^3^, Stuart Dowall^1^, Stephen Findlay-Wilson^1^, Victoria Graham^1^, Kuiama Lewandowski^1^, Daniel P. Carter^1^ and Roger Hewson^1,4^

^1^National Infection Service, Public Health England, Porton Down, SP4 0JG, United Kingdom

^2^Centre for Chemical, Radiation and Environmental Hazards, Public Health England, Chilton, OX11 0RQ, United Kingdom

^3^CBR Division, Defense and Science Technology Laboratories, Porton Down, SP4 0JG, United Kingdom

^4^Faculty of Infectious Tropical Disease, London School of Hygiene & Tropical Medicine, United Kingdom

Corresponding author: Babak Afrough

Email: [Babak.Afrough@gov.uk](mailto:Babak.Afrough@gov.uk)

**Supplementary data**

**Monte Carlo dose maps.** Preliminary MC simulations were benchmarked against IC dose rate data, where limited locational data was generated for comparison against corresponding measurements. The full spatial dose map within the irradiation chamber was next considered. This was achieved by defining a 20 x 20 grid of 1 x 1 x 1 cm^3^ cubes across the irradiation field, each filled with water. The entire lattice was then surrounded by a 0.2 cm thickness of polypropylene, itself encased in 0.0228 cm of polyethylene, respectively representing the walls of the specimen packaging in the real scenario; these layers of plastic were assumed sufficient to reproduce the correct attenuation of the direct and backscattered components of the field impinging on the samples, with its lateral components assumed dosimetrically negligible.

A


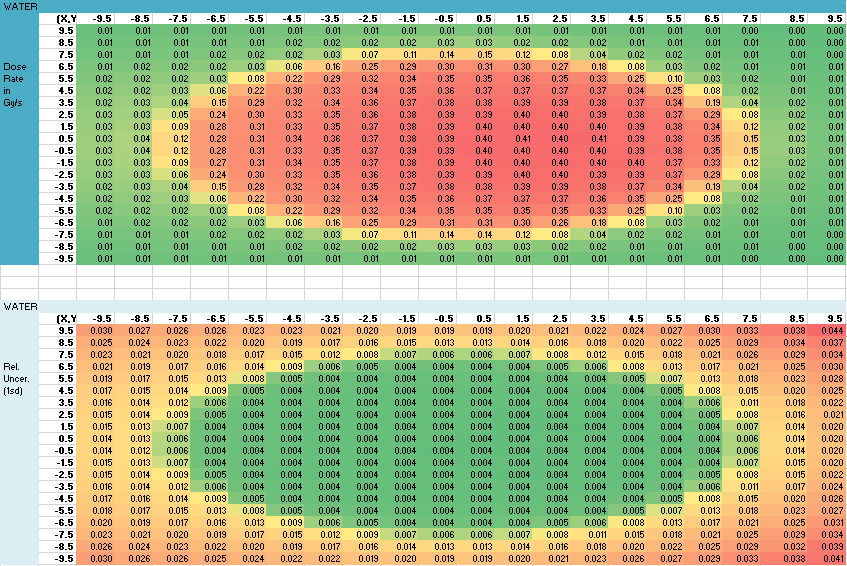


B


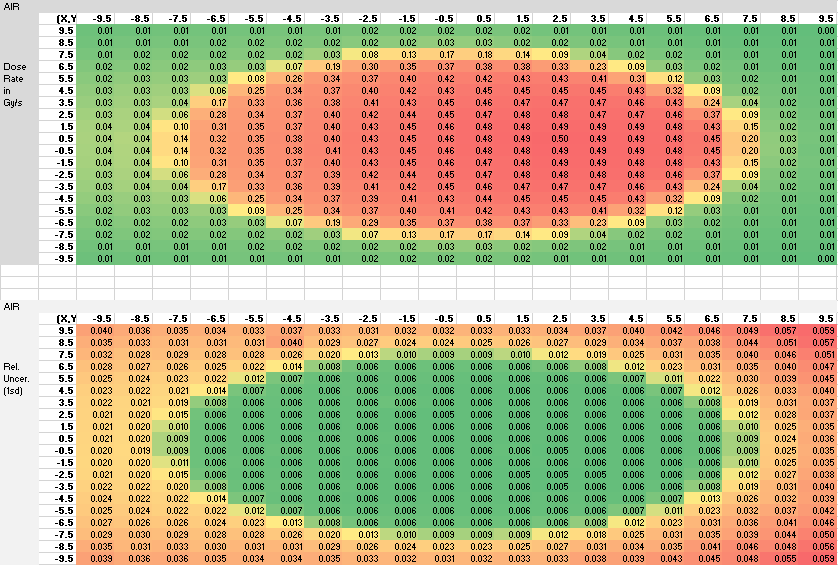


C


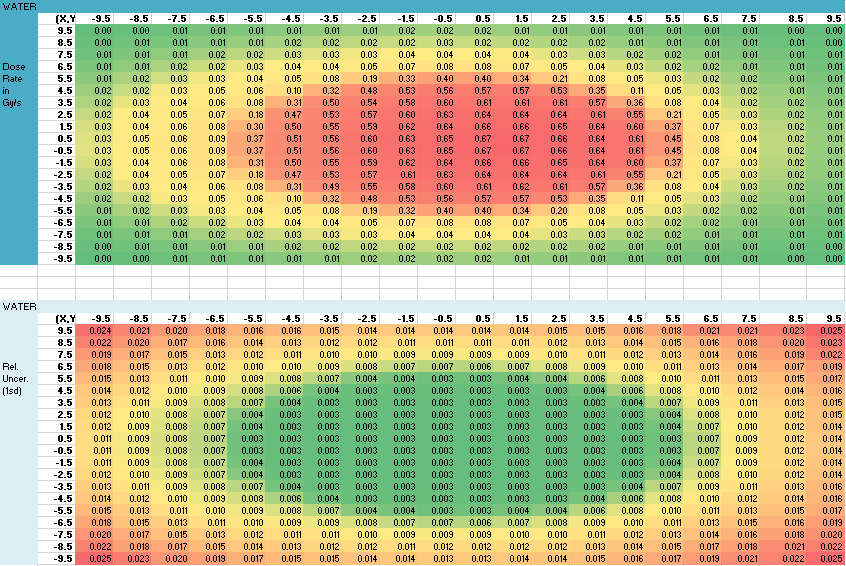


Figure S1. Predicted Monte Carlo dose maps for 0.2 mm Al filtration. *(a) On-shelf dose rates to water (Gy/s) and (b) on-shelf air kerma rates (Gy/s) for the 0.2 mm Al filtered field at position (x,y) for shelf at z= 22.6 cm, for 220 kV tube potential and 18.2 mA current and (c) at 220 kV, 18.2 mA at SDS 16.5 cm; in each pair, the lower figure shows the relative 1 standard uncertainty from the Monte Carlo calculations, which in all cases are seen to be low. The ratios of (16.5*16.5)/(22.6*22.6) is 0.533, which is very close to the ratio of (0.34/0.65) (=0.523) between the dose rates at the central locations irrespective of distance i.e. confirming that the dose rate is roughly scalable as the inverse square of the distance from the source, as it would be broadly expected. Therefore, the diameter of the central 'hot' region will also likely scale similarly geometrically.*

Thus, the modelled configuration was essentially equivalent to exposing 400 samples simultaneously, each of volume 1 ml and each occupying a unique 1 cm^2^ location within the ranges -10 ≤ *x* ≤ 10 and 10 ≤ *y* ≤ 10. The average kerma to each volume was then calculated individually by MCNP: a spatial map of dose deposition to water as a function of (*x*,*y*) position was thus obtainable. A corresponding air kerma map was also obtained in a similar way, but with the input file modified such that all relevant volumes were instead filled with air. Figures S1 show predicted dose rates in (a) air, (b) water and (c) proportional scaling as expected via the inverse square of the distance law pertinent to scaling, along with the relative 1 standard uncertainty on each data point relating to the statistical variation within the Monte Carlo models.

In addition to the dose rate being broadly constant across the area covering the biological sample (Figure S1), it is important also to ensure that the incident X-ray field is similarly uniform. Figures S2 show the free-in-air fluence-energy distribution of the photons incident on the grid as a function of lateral location. The data were essentially generated by defining binned (1 keV resolution) ‘*f4:p*’ fluence tallies across volumes centered at 1 cm radial increments on both the ± *x-* and +*y-*axes, the latter positive truncation being justified from the electron-beam symmetry within the X-ray tube itself. Results for the unfiltered field are shown because that configuration can be expected to show the greatest variation with position, but analogous data may be provided for the filtered fields. However, it is demonstrated that the spectra are highly similar across the ranges ⎥*x*⎢ ≤ 7.5 cm and *y* ≤ 7.5 cm, as anticipated from the beam cones indicated in Figures S1. Beyond these ranges, the greater contribution from back-scattered photons relative to the decreasing fluence of primary photons can be shown (Figure S1 (*top left*); analogous results for *x* >7.5 cm are omitted for brevity) to perturb the fields significantly, but of course this is not deleterious to the sample exposures of relevance to the current work.

**Figure S2. Photon energy spectra as a function of lateral position**. *(Top left) For y ≤ 7.5 cm and x=0; (Top right) For y ≤ 9.5 cm and x=0; (Bottom left) For 0<x ≤ 7.5 cm and y=0; (Bottom right) For ‑7.5≤x <0 cm and y=0. Data are shown for the unfiltered field for shelf at z= 22.6 cm, for 220 kV tube potential.*

**X-ray beam dose linearity.** To address the error rate across the targeted sample radiation field a dual approach of direct measurements in air using ionization chamber and Monte Carlo (MC) predictions in liquid phase (water) at standard room temperature were adopted. Absorbed dose (Gy) data was measured using a type TN31010 Semiflex ionization chamber (IC) connected to a UNIDOS E electrometer. These measurements were made at selected locations within a defined geometric radiation field with the X-ray source set to 18.2 mA, 220 KeV. This targeted radiation field (*Ø*) produced a surface area of 81 cm^2^ (9 cm x 9 cm) defined by *x*, *y*, and *z* Cartesian coordinates at 22.6 cm from the source. MC simulation data for these conditions were developed in parallel to model and quantify uncertainty and dose exposure variability within a packaged specimen under physiological conditions.


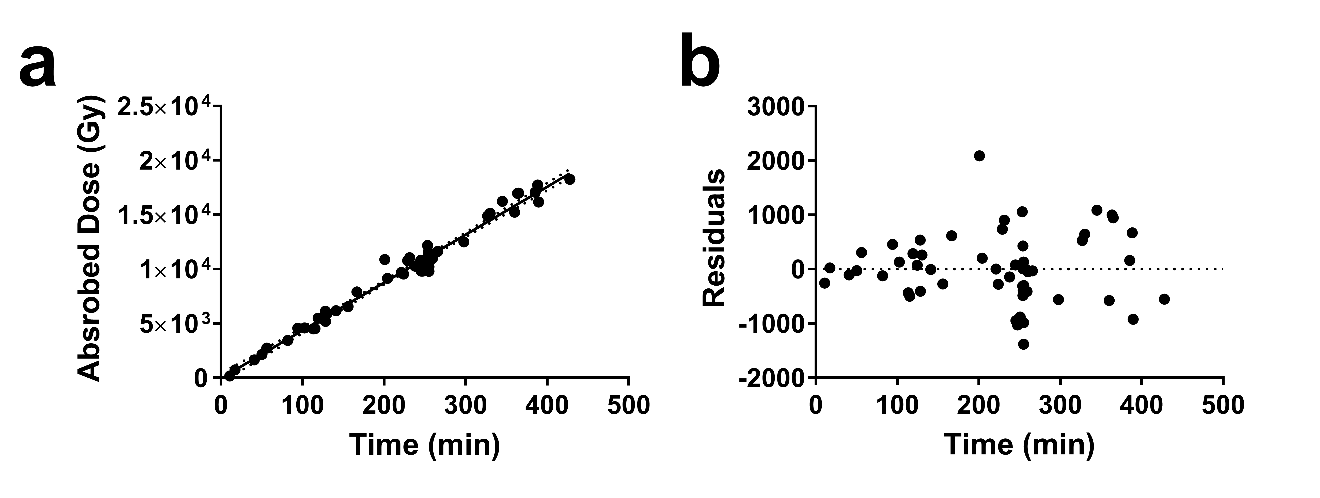


Figure S3. Radiation dose vs time. *(a) regression analysis of real-time absorbed dose measured using a TN31010 Semiflex ionization chamber across 56 independent timepoints during continuous cycles of X-ray irradiation. The R^2^ value is 0.9804 with -460.2 to 369.1 95% CI for the dose variable. (b) residuals of the linear regression model showing random heteroscedasticity around the mean.*

By accounting for dose rates at fixed Cartesian positions, we show estimates of absorbed dose (Gy/s) and photon energies across the targeted geometric field for a range of filters. The relationship between absorbed dose and time was determined using paired observations of real-time IC readings across 56 independent experiments and demonstrates a linear relationship (Figure S3 A). The data points used in this analysis represent randomized Cartesian coordinates of the targeted sample radiation field. The root mean square error of this regression model predicts ± 206.8 Gy variance over time, however the residuals of this model shows an increasing trend in variance of dose over time (Figure S3 B).

**ZIKV Envelope Western blot.** Western blot images used in Figure 3d of the manuscript has been edited to include additional experiment details of relevance. Here the original unprocessed image is presented. For gel annotation please refer to Figure 3d of the manuscript.

**
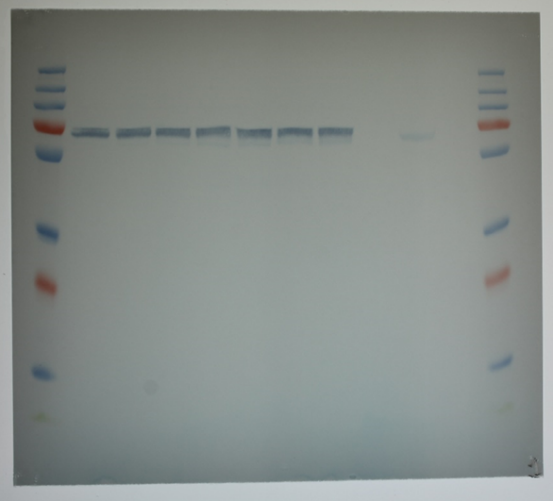
**

**Figure S4. Detection of X-ray inactivated ZIKV.** *Concentration of cell culture derived ZIKV and control samples using 100 kDa MWCO Amicon Ultra-0.5 Centrifugal filters cat code: UFC5010 (Millipore, UK) were subjected to denaturation at 95˚C for 10 min under reducing conditions. Following gel electrophoresis, protein was transferred on to a PVDV membrane (using a standard wet transfer methods) and probed with a Rabbit derived polyclonal anti-ZIKV envelope IgG, cat code GTX133314 (Genetex, USA). The molecular weight marker is PageRuler Plus prestained protein ladder, 10 to 250 kDa (Thermo Scientific, UK) showing bands in the appropriate lanes.*
